# Breaking Bad News, a Pertinent Yet Still an Overlooked Skill: An International Survey Study

**DOI:** 10.3390/healthcare8040501

**Published:** 2020-11-20

**Authors:** Abbas Alshami, Steven Douedi, America Avila-Ariyoshi, Mohammed Alazzawi, Swapnil Patel, Sharon Einav, Salim Surani, Joseph Varon

**Affiliations:** 1 Department of Medicine, Jersey Shore University Medical Center, Neptune, NJ 07753, USA; abbas.alshami@hackensackmeridian.org (A.A.); steven.douedi@hackensackmeridian.org (S.D.); mohammed.alazzawi@hackensackmeridian.org (M.A.); swapnil.patel@hackensackmeridian.org (S.P.); 2 Research Department, Dorrington Medical Associates, Houston, TX 77030, USA; america_aa@hotmail.com; 3 Shaare Zedek Medical Center, Jerusalem 9103102, Israel; einav_s@szmc.org.il; 4 Department of Medicine, Texas A&M University, Corpus Christi, TX 77843, USA; srsurani@hotmail.com; 5 Department of Pulmonary and Critical Care, The University of Texas Health Science Center at Houston, Houston, TX 77030, USA

**Keywords:** ethical issues, intensive care units, communication, truth disclosure, life change events, physician-patient relations

## Abstract

Delivering bad news to patients is a challenging yet impactful everyday task in clinical practice. Ideally, healthcare practitioners should receive formal training in implementing these protocols, practice in simulation environments, and real-time supervision with feedback. We aimed to investigate whether healthcare providers involved in delivering bad news have indeed received formal training to do so. We conducted a cross-sectional survey study that targeted all healthcare providers in the intensive care units of 174 institutions in 40 different countries. Participants included physicians, nurses, medical students, nursing students, pharmacists, respiratory technicians, and others. The survey tool was created, validated, and translated to the primary languages of these countries to overcome language barriers. A total of 10,106 surveys were collected. Only one third of participants indicated that they had received a formal training. Providers who had received formal training were more likely to deliver bad news than those who had not. Younger and less experienced providers tend to deliver bad news more than older, more experienced providers. The percentage of medical students who claimed they deliver bad news was comparable to that of physicians. Medical schools and post-graduate training programs are strongly encouraged to tackle this gap in medical education.

## 1. Introduction

Historically, medical education focused merely on technical proficiency and often neglected communication skills [1]. While this approach may have been appropriate when doctor–patient relationships were paternalistic, it is no longer acceptable in the age of patient autonomy. The introduction of the biopsychosocial model by Engel four decades ago changed the way we care for patients, emphasizing the psychosocial aspects of care [2]. Among these, a crucial skill is the ability to deliver bad news.

Delivering bad news to patients and/or families of patients is a challenging yet almost everyday task in clinical practice. In oncology and intensive care, the burden of such communication can be overwhelming. Providers finding it difficult to deliver bad news engender greater patient dissatisfaction and have poorer patient outcomes [3,4]. Failed communication can also lead to loss of trust and even litigation [5]. Therefore, a careful approach should be adopted when conducting such conversations [6,7].

Several authors have proposed protocols for delivery of bad news [8,9,10]. These protocols outline the steps providers should take to minimize the impact of such news on the patient and his/her family. Studies have shown that teaching and training in the use of such protocols can improve provider skills, thereby also improving patient outcomes [11,12]. Ideally, healthcare providers should be taught these methods and undergo training in the use of such protocols. We sought to investigate whether healthcare providers who deliver bad news to patients and families had indeed received formal education on how to do so.

## 2. Materials and Methods

The ETHICS study was a cross-sectional survey conducted across five continents by direct (face-to-face) approach to clinicians [13,14]. Participant selection was based on convenience sampling following receipt of agreement to use the anonymous data collected for publication in a professional journal. The study was conducted in multiple countries with differing Institutional Review Board (IRB) requirements. As no data were collected on patients, the questionnaires were filled anonymously, the relevant data from the participating clinicians were only their demographics, education, and opinions, and it was clarified before questionnaire completion that participation constitutes consent to use the data for publication, formal IRB approvals were not sought.

### 2.1. Participants

The ETHICS survey targeted healthcare providers directly involved in patient care in intensive care units (ICUs), including physicians, nurses, pharmacists, technicians, and even students performing clerkships. We accepted that an ICU is indeed such if it was viewed as such within the respective institution. We aimed to survey the entire population of healthcare providers working in ICUs. Participants completed the questionnaire only after clarification that this constitutes informed consent for use of their anonymized data for research purposes.

### 2.2. Development of the Assessment Tool

#### 2.2.1. Creation

The questionnaire was created by the research team after extensive literature review for pertinent studies and several discussions with healthcare providers, religious figures, and researchers. The questionnaire, constructed specifically for the purpose of the ETHICS study, consisted of 30 questions, including demographic information and questions on ethical issues frequently encountered in the practice of critical care. The questionnaire included both open questions (free text answers) and closed questions (dichotomous and nominal items). The questions were selected, and their wording agreed on by the research team as a whole.

#### 2.2.2. Validation

Two experts in critical care medicine reviewed the questions to ensure adequacy, relevance, and clarity (e.g., face validity). Then, a priest reviewed the wording of the questionnaire to ensure the appropriateness of the language used. The consistency of the tool was then assessed in a pilot study conducted on 20 healthcare providers, who answered the questionnaire on two occasions, two weeks apart, to ensure adequate intra-rater reliability. Intraclass correlation coefficient (ICC) using a two-way mixed-effects model, average measurement type, and absolute agreement definition were used to assess the reliability of continuous variables [15]. Fleiss Kappa (κ) coefficient (using SPSS extension named “STATS_FLEISS_KAPPA” by David Nicholas v1.1.1) was used for categorical variables. As this was an exploratory study, an ICC > 0.5 and a κ > 0.7 were considered adequate for question inclusion in the final version of the questionnaire.

#### 2.2.3. Translation

The questionnaire was prepared in the English language and underwent translation to several languages: Spanish, Arabic, Japanese, Chinese, Bahasa Indonesia, Polish, German, Portuguese, and Hebrew in three stages. First, the questionnaire was translated to each target language by two independent translators (forward translation). Discrepancies between the two translations were resolved by discussion and agreement. Second, a third translator, independent of the other two translators, translated back the questionnaire to the English language. Finally, an expert committee formed by the primary investigator and two other experts, the three translators, and two researchers involved in creating the original questionnaire reviewed all the versions of translations and determined the final version for each language. The final translated questionnaires did not undergo further validation.

### 2.3. Data Collection

The principal collaborator in each institution administered the surveys and, once completed, mailed them back to the Principal Investigator (J.V.). Participants were reminded three times (once weekly) to complete their surveys and return them to a locked box. After the third week, each collaborator removed the locked box and shipped the collected surveys by mail to the aforementioned study site.

### 2.4. Outcomes

The primary outcome measure was the percentage of physicians who had received formal training on delivery of bad news. The secondary outcome measures included were the relation of age and years of experience with delivery of bad news.

### 2.5. Data Analysis

Data were analyzed using IBM SPSS Statistics TM version 26.0 (IBM Corporation, Artmonk, NY, USA). The Shapiro–Wilk test was used to assess for normality of continuous variables. The SPSS package for missing value analysis was used to determine the pattern of missing data, and binary logistic regression was used to determine whether missing data were missing complete at random (MCAR), missing at random (MAR), or missing not at random (MNAR). Then, multiple imputation with the Makarov Mccain Monte Carlo model (MCMC) was used to create a total of 5 imputations with 20 iterations, which provided sufficient convergence [16,17]. Auxiliary variables, identified by logistic regression modeling, were also sought and included in the imputation model to increase estimation precision.

Descriptive analyses were used for the study questions, and the results were stratified by demographic variables. The Chi-square test was used to compare categorical variables. The Mann–Whitney U test was used to compare continuous non-parametric variables with estimation of effect size (r) [18]. The independent-sample Kolmogorov–Smirnov (K–S) test was used to compare variables with non-parametric distributions. In addition, variables that correlated in univariate analysis with study questions were entered in a multivariable logistic regression (enter method). Correlation matrix was created and examined to assess for any potential multicollinearity. Concordance statistic (c statistic) and Hosmer–Lemeshow (HL) test were calculated to assess the discriminative ability and goodness of fit of the models, respectively. An alpha (*p*) value of 0.05 was used to ascertain statistical significance. Two questions were used to determine whether providers had delivered bad news before and whether they had received formal training prior to doing so.

## 3. Results

**Figure 1:**
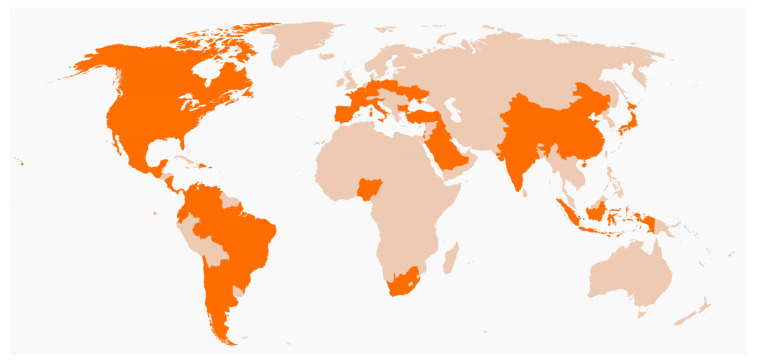
World map depicting countries where the study was conducted in in orange.

A total of 13,681 surveys were distributed, and a total of 10,106 surveys were returned (response rate 73.86%). Participants from the ICUs of 174 institutions in 40 different countries completed the survey (see Figure 1). Around 58% of the ICUs were of academic institutions *(n* = 101), and 42% were for community-based institutions (*n* = 73).

### 3.1. Reliability Measures

For categorical variables, the Fleiss kappa coefficient was 0.84 (95% CI 0.76–0.92, *p* < 0.001), indicating good to very good reliability [19]. ICC for the only continuous variable was 0.917 (95% CI 0.825–0.961, *p* < 0.001), indicating good reliability as well [15].

### 3.2. Missing Data

Overall, 68% (*n* = 6867) of surveys were complete, while 32% (*n* = 3239) missed at least one value. Of the total number of values in the data pertinent to this study, 95.57% (*n* = 86,925) were reported, and 4.43% were missing (*n* = 4029). Only 1.74% (*n* = 176/10,106) of the respondents did not complete the question regarding whether they had received formal training in delivering bad news. There was a statistically significant correlation between a lack of response to this question and lack of data on sex, profession, and the city. However, logistic regression modeling (enter method) with these three independent variables associated only lack of data regarding sex and profession with lack of data regarding training. Overall, 9.64% (*n* = 974/10,106) of the respondents did not complete the question regarding whether they had delivered bad news before. There was a statistically significant correlation between a lack of response to this question and lack of data on sex, age, profession, city, and previous formal training in delivering bad news. Logistic regression modeling with these five independent variables associated all these variables except age with lack of data regarding training.

### 3.3. Demographic Data

Among the participants who completed the questionnaires, 41% (*n* = 4142) were male and 59% (*n* = 5964) were females. In terms of profession, 30.8% (*n* = 3111) were physicians, 22.6% (*n* = 2281) were nurses, 28.7% (*n* = 2921) were medical students, and 17.7% (*n* = 1793) stated were allied health professionals (e.g., pharmacists, respiratory technicians, and nursing students). Despite the large number of participants, participant age and years of experience both followed non-normal distributions (Shapiro–Wilk test). The mean age of the participants was 31.63 years (95% CI 31.41–31.86 years), median 28 years, 25th–75th interquartile range was 22–38 years, and range was 18–88 years. For years of experience, the mean was 9.04 years (95% CI 8.85–9.22 years), median was 5 years, 25th–75th interquartile range was 2–14 years, and range was 0–55 years.

### 3.4. Response to the Question “Have You Received Formal Training on How to Deliver Bad News?”

Only 33.4% (95% CI 32.5–34.3%) of the respondents reported having formal training on how to deliver bad news (*n* = 3376). The rate of training was low in all professions but did somewhat differ between professions (40.2% in physicians (*n* = 1251), 37.4% of nurses (*n* = 852), and medical students 26.6% (*n* = 778), 95% CI 38.44–41.96%, 35.41–39.38%, and 25–28.2%, respectively, *p* < 0.001). Providers who had received formal training on delivering bad news were more likely to do so themselves than those who did not (74.6% (2518/3375) vs 66.6% (4484/6731), *p* < 0.001, 95% CI 73.1–76.1%). Respondents who had received formal training were older (mean rank 5465 vs. 4847, r = 0.1) and had more years of experience (mean rank 5494 vs. 4832, r = 0.11). Direct comparisons between means or medians for age or years of experience were not feasible, as these followed different distributions. In addition, age, gender, profession, years of experience, continents, and type of institution (academic vs. community) were initially entered a logistic regression model. However, age was then removed due to high multicollinearity with years of experience (absolute correlation coefficient 0.86). Results are shown in Table 1. C statistic was 0.6 ± 0.006, and HL showed good fit of model (*p* = 0.55).

### 3.5. Response to the Question “When a Patient under Your Care Dies, Who Would Deliver the News to the Family?”

Overall, 69.3% of the respondents reported “myself” as the answer to this question (*n* = 7006), while 30.7% would leave the delivery of the bad news to someone else (*n* = 3100). Physicians and medical students were more likely to deliver bad news than nurses (88.2% (*n* = 2744, 95% CI 87.1–89.3%) and 82.6% (*n* = 2413, 95% CI 81.2–84%) vs. 40.4% (*n* = 922, 95% CI 38.4–42.4%), respectively (*p* < 0.001)).

Providers who deliver bad news themselves tend to be younger (mean rank 4810 vs. 5600, r = 0.12, *p* < 0.001) and less experienced (mean rank 4861 vs. 5488, respectively, r = 0.1, *p* < 0.001) than those who do not. Direct comparisons between means or medians for age or years of experience were not feasible, as these followed different distributions. In addition, gender, profession, years of experience, continent, type of institution, and previous formal training were inputted in a multivariable logistic regression model to further determine the factors independently associated with leaving delivery of bad news to someone else (Table 2). C statistic was 0.76 ± 0.005, and HL test showed good fit of the model (*p* = 0.38).

## 4. Discussion

This study surveyed more than 10,000 clinicians that treat critically ill patients globally and found that only one third of these had received training on delivering bad news. We also found that younger healthcare workers and those with fewer years of work experience had less formal training yet were more likely to be involved in delivering bad news (though effect sizes were small). We also found that providers in Asia and Europe were less likely to report previous formal training and more likely to report leaving delivery of bad news to someone else; however, we would interpret these results with caution, as adequate sampling to assure generalizability at continent level was not ensured. Moreover, providers in academic institutions were more likely to report receiving formal training and more likely to leave delivery of bad news to someone else.

Our results are supported by the findings of a single center survey study conducted in 2016, which reported that 43% of physicians had received training in delivering bad news (40.2% of physicians in our study) [1]. In 2000, Baile et al. reported that less than 10% of physicians had received formal training on delivering bad news [10]. Given the number of years that elapsed between these two studies, we may assume that a greater number of medical educational systems are incorporating training on delivering bad news in their curricula, reflecting increased awareness of this need within healthcare. Nonetheless, the percentage of healthcare providers formally trained remains modest relative to the anticipated need.

The fact that the percentage of physicians trained in delivering bad news is higher than that of medical students (40.2% vs. 26.6%) could either indicate very late training in medical schools or that many physicians are receiving such training only after graduation from medical schools. This is particularly important in light of our finding of an almost comparable percentage of physicians and medical students who claim they deliver bad news themselves (88% vs. 82%). Educational programs on delivery of bad news and communication skills in general should be implemented in the earliest possible stage of medical education. In addition, medical students should be taught to refrain from delivery of bad news unless they have undergone formal training, preferably including simulation scenarios, and should be encouraged to do so under adequate supervision.

Our finding regarding greater involvement of younger healthcare workers with fewer years of work experience in delivery of bad news probably reflects the greater administrative workload of older clinicians. We did not study whether the less experienced providers who claim they deliver bad news themselves are supervised at this time. However, Orlander et al. surveyed 129 medical residents, and these reported that, when they had delivered bad news, a senior resident or attending physician was present during a mere 11% and 5% of their first clearly remembered such encounters [20].

Several institutions have studied the effects of incorporating training programs on delivery of bad news in their curricula. One such program used the “SPIKES” protocol and included simulated patient exercises [21,22]. The program increased the self-reported preparedness of fourth year medical students to convey bad news to patients from 63.4% prior to training to 93.7% after completion of training [22]. Although we did not explore the causes for leaving delivery of bad news to others in our study, increased preparedness with training, reported in other studies, may explain our finding of lower likelihood of leaving delivery of bad news to others in providers with previous training (OR 0.64). Formal training with simulation experiences should ideally be implemented in every medical training program, combined with real-time observation and feedback during the early years of practice. Otherwise, patients or their families may continue to suffer the ramifications of poor delivery of bad news, such as depression, anxiety, and non-adherence, which may last for long time [7,23].

We pre-identified several limitations during the study design stage. It is imperative to understand that cross-sectional design of the study does not allow for adequate assessment of causality. Additionally, the sampling process was done by convenience (non-probability), and there was no consistent method of identifying the entire population of providers in ICUs. These factors can lead to selection bias. In addition, direct validation of the surveys translated to non-English languages was not done. Furthermore, we could not explore the characteristics of non-respondents. Moreover, we could not evaluate internal consistency using statistical measures since no more than one question measured the same construct. This decision was taken knowingly in order to encourage a relatively high response rate. In addition, the aim was to conduct an exploratory rather than a confirmatory study; for which the current design was felt to be adequate. Additional potential biases include but are not limited to self-reporting bias (both social desirability and recall bias) and administration bias as participants were involved in the survey collection process. Moreover, we acknowledge that one of the logistic regression models does not have a good discriminative ability (c statistic = 0.6), however, it was considered adequate since the aim was to explore the associations rather than to make a prediction model.

## 5. Conclusions

Despite the potentially long-lasting sequalae of poor delivery of bad news on patients and their families, it seems that a large part of the clinicians treating critically ill patients globally have never received training on delivering bad news. Medical schools and post-graduate training programs are strongly encouraged to tackle this gap in medical education.

## Figures and Tables

**Table 1:** Factors associated with reporting previous formal training in a multivariable logistic regression model.

Variable	Crude OR	95% CI	*p* Value	Adjusted OR	95% CI	*p* Value
Years of Experience	1.023	1.018–1.027	<0.001	1.013	1.008–1.018	<0.001
Sex (male)	1.116	1.026–1.213	0.01	1.076	0.985–1.176	0.10
Profession (Physician)	1		Ref	1		Ref
Profession (Nurse)	0.893	0.798–1	0.05	0.927	0.824–1.044	0.21
Profession (Student)	0.542	0.486–0.605	<0.001	0.593	0.522–0.675	<0.001
Profession (Allied)	0.532	0.469–0.604	<0.001	0.568	0.498–0.648	<0.001
Continent (South America)	1		Ref	1		Ref
Continent (Europe)	0.855	0.695–1.052	0.14	0.797	0.646–0.983	0.03
Continent (North America)	0.909	0.769–1.075	0.27	0.864	0.729–1.025	0.09
Continent (Asia)	0.853	0.701–1.039	0.12	0.759	0.622–0.927	0.007
Continent (Africa)	1.111	0.715–1.724	0.64	1.194	0.765–1.864	0.44
Institution Type (Academic)	0.501	0.456–0.550	<0.001	1.187	1.063–1.327	0.002

**Table 2:** Factors associated with reporting leaving breaking bad news to someone else in a multivariable logistic regression model.

Variable	Crude OR	95% CI	*p* Value	Adjusted OR	95% CI	*p* Value
Years of Experience	1.013	1.009–1.018	<0.001	1.013	1.007–1.019	<0.001
Sex (male)	0.545	0.498–0.595	<0.001	0.887	0.801–0.982	0.02
Profession (Physician)	1		Ref	1		Ref
Profession (Nurse)	10.96	9.52–12.62	<0.001	10.75	9.28–12.46	<0.001
Profession (Student)	1.566	1.345–1.823	<0.001	1.643	1.384–1.951	<0.001
Profession (Allied)	6.928	5.978–8.027	<0.001	6.864	5.870–8.026	<0.001
Continent (South America)	1		Ref	1		Ref
Continent (Europe)	0.987	0.791–1.231	0.91	1.141	0.891–1.461	0.30
Continent (North America)	0.897	0.751–1.072	0.23	1.039	0.852–1.268	0.70
Continent (Asia)	1.239	1.008–1.524	0.04	1.264	1–1.597	0.05
Continent (Africa)	0.224	0.110–0.453	<0.001	0.455	0.217–0.955	0.04
Institution Type (Academic)	1.041	0.933–1.163	0.47	1.154	1.014–1.314	0.03
Previous Formal Training	0.499	0.474–0.526	<0.001	0.640	0.572–0.715	<0.001
